# Development of Colloidal Gold-Based Immunochromatographic Assay for Rapid Detection of Goose Parvovirus

**DOI:** 10.3389/fmicb.2018.00953

**Published:** 2018-05-14

**Authors:** Xianglong Yu, Lei Wei, Hao Chen, Xiaoyu Niu, Yanguo Dou, Jing Yang, Zhenzhong Wang, Yi Tang, Youxiang Diao

**Affiliations:** ^1^College of Animal Science and Technology, Shandong Agricultural University, Tai’an, China; ^2^Shandong Provincial Key Laboratory of Animal Biotechnology and Disease Control and Prevention, Tai’an, China; ^3^Shandong Provincial Engineering Technology Research Center of Animal Disease Control and Prevention, Tai’an, China; ^4^Tai’an City Central Hospital, Tai’an, China

**Keywords:** goose parvovirus, colloidal gold, immunochromatographic strip, monoclonal antibody, rapid detection

## Abstract

Goose parvovirus (GPV) remains as a worldwide problem in goose industry. For this reason, it is necessary to develop a new diagnostic approach that is easier and faster than conventional tests. A rapid immunochromatographic assay based on antibody colloidal gold nanoparticles specific to GPV was developed for the detection of GPV in goose allantoic fluid and supernatant of tissue homogenate. The monoclonal antibodies (Mab) was produced by immunizing the BALB/c mice with purified GPV suspension, and the polyclonal antibody (pAb) was produced by immunizing the rabbits with recombinant VP3 protein. The colloidal gold was prepared by the reduction of gold salt with sodium citrate coupled with Mab against GPV. The optimal concentrations of the coating antibody and capture antibody were determined to be 1.6 mg/ml and 9 μg/ml. With visual observation, the lower limit was found to be around 1.2 μg/ml. Common diseases of goose were tested to evaluate the specificity of the immune colloidal gold (ICG) strip, and no cross-reaction was observed. The clinical detection was examined by carrying out the ICG strip test with 92 samples and comparing the results of these tests with those obtained via agar diffusion test and polymerase chain reaction (PCR) test. Therefore, the ICG strip test was a sufficiently sensitive and accurate detection method for a rapid screening of GPV.

## Introduction

Goose parvovirus (GPV) are small, non-enveloped, single-stranded DNA viruses, which have been classified in the family parvoviridae, genus parvovirus. The GPV particles can be observed in hexagonal or circular appearance with a diameter of 20–24 nm through scanning electron microscope. Three kinds of structural proteins (VP1, VP2, VP3) present in the virus, wherein VP3 protein is the major structural protein (Shao et al., 2014). This virus was first detected by Fang (1962) in China. In the following years, GPV was reported in Europe. GPV infection is caused by GPV, which is an acute or subacute sepsis in 4–20 days old goslings characterized by high infectiousness and exudative inflammatory bowel disease. This disease spreads rapidly and has a high fatality rate, and the mortality rate of goslings within 5 days old is above 95%. The transmission and prevalence of GPV infection has caused severe economic losses and restricted the development of goose industry.

The current methods for diagnosing GPV infections are routine molecular assays such as polymerase chain reaction (PCR) test (Limn et al., 1996), fluorescent quantitative real-time PCR test (Yang et al., 2009), agar diffusion test and Loop-Mediated isothermal amplification (Yang et al., 2010). Indirect ELISA based on VP3 proteins are commonly used to detect GPV antibodies (Zhang et al., 2010). All of these are effective and accurate methods of detecting viral infections, however, these methods are time consuming and instrument required, and can only be carried out in laboratories by professional. Therefore, development of a rapid detection method to monitor GPV would be desirable.

The one-step immunochromatographic assay using gold nanoparticles has been widely used in certain fields (Meng et al., 2014). This method is rapid and convenient to use and requires few equipment. However, there is no report about the detection of GPV using the one-step immunochromatographic assay. So, the aim of this study was to establish a colloidal gold-based immunochromatographic assay for the rapid detection of GPV, to control the spread and epidemic of the disease, and to promote the development of goose industry.

## Materials and Methods

### Ethics Statement

All applicable international, national, and institutional guidelines for the care and use of animals were followed to minimize suffering. Animal procedures was approved by the Committee on the Ethics of Animal of Shandong (permit number: 201750017). Two young adult (35-day-old) BALB/c mice were used in the immunization protocols and maintained at Shandong Agricultural University with water and food *ad libitum*, relative humidity 40–60% conditions and a 12–12 h light-dark cycle. Mice were euthanized using rapid cervical dislocation.

### Materials and Reagents

Goose parvovirus, avian influenza virus (H5, H7, H9), duck hepatitis virus, tembusu virus, fowl adenovirus, goose reovirus and muscovy duck parvovirus were isolated and maintained in our laboratory. The purified GPV was isolated from the GPV-positive allantoic fluid by sucrose density gradient centrifugation in our laboratory. Recombinant VP3 protein was expressed by prokaryotic expression system in the previous work and VP3 concentration was determined to be 3.34 mg/ml by the BCA Protein Assay Kit. Goat anti-mouse IgG antibody and bovine serum albumin (BSA) were purchased from Boster Biological Technology Co., Ltd (Wuhan, China). Nitrocellulose (NC) membrane, absorbent pad, sample pad, conjugate pad, and PVC sheets were obtained from Millipore (Shanghai, China). Hydrogen tetra-chloroaurate hydrate (HAuCl4) and trisodium citrate were obtained from Shanghai Chemical Reagents (Shanghai, China). Phosphate buffer saline (PBS, pH 7.4, 0.01M in 0.85% NaCl) was prepared in our lab. BALB/c mice were purchased from Spfanimals, Inc. (Beijing, China). All other chemicals in the present study were either chemical pure or with highest quality.

### Preparation of Monoclonal Antibodies

Monoclonal antibodies against GPV (anti-GPV Mab) were prepared as described by Yin et al. (2010) with some modifications. Briefly, purified GPV suspension was verified by PCR test and analyzed by SDS-PAGE, then emulsified with Freund’s adjuvant in a 1:1 ratio and BALB/c mice were immunized with the fluid at the volume of 0.2 ml. Hybridomas were generated by the fusion of spleen cells to SP2/0 myeloma cells. The reactivity and specificity of hybridoma cells to GPV were tested by Indirect ELISA. For Indirect ELISA, 2 μg/ml purified GPV in carbonate buffer solution was incubated in black Maxisorb 96-well plates overnight at 4°C and the plates were blocked by 5% non-fat dry milk for 2 h at 37°C, then supernatant of hybridoma was then added at 100 μL/well. Anti-GPV Mab was selected through three successive limiting dilutions, and supernatants of hybridoma showing strong reactivity against GPV were used for mass production of Mabs. Ascites containing abundant Mabs were obtained from big female BALB/c mice, and purified by affinity chromatography (Shukla and Thommes, 2010). The concentration of the Mabs was determined with the DeNovix microvolume spectrophotometer (DeNovix, Inc., DS-11, United States), and used as a reference for optimal concentration of the coating antibody.

### Preparation of Polyclonal Antibody

Polyclonal antiserum against GPV were prepared according to a previously published report with slight modifications (Ma et al., 2017). It was generated in rabbits by immunizing the animals with recombinant VP3 protein and used as a coating reagent fixed on NC membrane (test line). Specific steps are as follows: VP3 protein was blended and emulsified with the same volume of FCA or FIA, then two healthy rabbits were injected into the back by multiple sites subcutaneous injection with the 200 μg VP3 protein. The entire immunization procedure comprised four injections, wherein the first immunization was with FCA, later with FIA. The first two injections were carried out at an interval of 2 weeks, and the subsequent two injections were carried out at intervals of 1 week. The polyclonal antibody was purified from the serum by sequential precipitation with caprylic acid and ammonium sulfate. Indirect ELISA was performed to measure the antibody titers of negative serum, unpurified serum and purified serum. The concentration of the antibodies was determined with Bradford method.

The pAb was diluted with PBS (0.01 M, pH 7.4) into gradient concentration (0.2 mg/ml, 0.4 mg/ml, 0.8 mg/ml, 1.6 mg/ml, 3.2 mg/ml), then fixed to the NC membrane with ZX1000 Dispense Platform (BioDot Inc., United States), and goat anti-rabbit antibody (Control line) was fixed simultaneously. The blank control and positive samples containing GPV were tested to determine the optimal concentrations of the coating antibody.

### Synthesis of Colloidal Gold

All glassware used, including beakers and flasks, was washed with ultrapure water, pretreated in aqua regia, then washed in ultrapure water and dried before use. Colloidal gold nanoparticles with a mean particle diameter of 20 nm were produced by reduction of gold chloride with 1% sodium citrate according to a previously described method (Zhang et al., 2015). Briefly, the ultrapure water (100 ml) was heated with electric heating to boiling point in an Erlenmeyer flask with a magnetic stirrer, and then 1 ml of the 1% the chloroauric acid solution were added to the flask rapidly, then 3.0 ml of 1% sodium citrate solution was added quickly into the solution under stirring. When the color of the solution changed from blue to dark red, the solution was boiled for another 10 min, then stopped heating, stirred for 15 min, then cooled and stored at 4°C with 0.05% sodium azide.

### Preparation of the Colloidal Gold–Mab Conjugate

The optimal amount of the Mab concentration for conjugation with the colloidal gold solution was determined at first (Liu et al., 2012). Briefly, the Mab was diluted with PBS (0.015 M, pH 7.4) into different concentration (0 μg/ml, 5 μg/ml, 7.5 μg/ml, 10 μg/ml, 20 μg/ml, 30 μg/ml, 50 μg/ml) and 20 μl solution of each concentration was added into 1 ml of colloidal gold solution (pH 8.5), with slight shaking for 10 min. Then 100 μl 10% NaCl was added into each tube and stirred for another 10 min. The mixtures were incubated for 1 h at room temperature and then observed. The color of the reaction changed from bright red to blue as the concentration of Mab decreases, and the optimum concentration of Mab for colloidal gold labeling is the minimum concentration of red invariant. Colloidal gold solution without NaCl was used as a negative control. Typically, the working concentration in the assay is 20% higher than the optimum concentration.

The colloidal gold probe was prepared according to the procedure in the literature with slight modification (Ju et al., 2010). The pH of the colloidal gold was adjusted to 8.5 by dropwise addition of 0.1 mol/L K_2_CO_3_. The Mab was diluted to 20 μl with PBS to the optimum concentration of Mab for colloidal gold labeling and added to 1 ml pH-adjusted colloidal gold solution, with stirring at intervals for 30 min. Then 20 μl of 10% BSA solution were added to the mixture to block the un-reacted sites of the gold nanoparticles, with stirring at intervals for 20 min, and then centrifuged at 12,000 rpm for 10 min. After centrifugation and removal of the supernatant, the precipitate was re-suspended in 100 μl dilution buffer [0.002 mol/L sodium carbonate solution (pH 8.5) containing 1% BSA and 0.2% sodium azide] and stored at 4°C for further use.

### Preparation of the Immunochromatographic Strip

The colloidal gold probe was added dropwise to the conjugate pad and dried under the fan for 2 h. Goat-anti-rabbit antibody and rabbit anti-GPV polyclonal antibody were fixed on a NC membrane at two discrete zones, with a volume of 1 μl/cm^2^ to form the test (T) line and control (C) line, respectively, with the Biodot equipment (BioDot, Inc., ZX 1000, United States).

**FIGURE 1:**
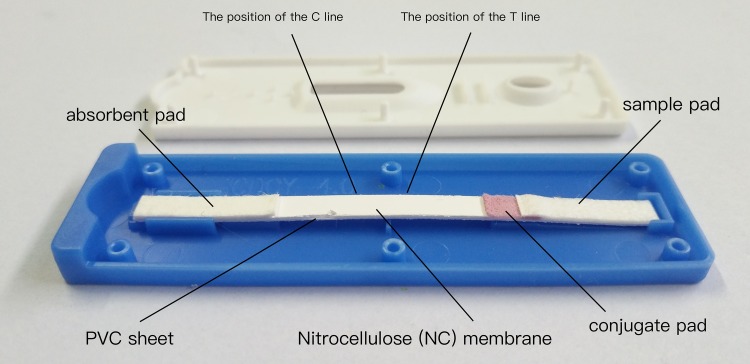
Schematic diagram of test strip.

The immunochromatographic strip consists five components (Liu et al., 2016): NC membrane, absorbent pad, sample pad, conjugate pad, and PVC sheets. The PVC sheet was used as the bottom of the test strip, the NC membrane pad was attached to the middle of the PVC sheet, the conjugate pad was attached to the edge of the membrane with 1–2 mm overlap, and then the sample pad was attached to the edge of the conjugate pad in a similar manner. The absorbent pad was attached to the top of the membrane with 1–2 mm overlap. The sheet was cut into cut into 4 mm-wide strips with a strip-cutter (model CM4000, BioDot, Irvine, CA, United States), and then sealed in a plastic backing plate. The schematic diagram of the test strip is shown in **Figure 1**. These strips were stored dried at 4°C for further use.

In order to ensure the accuracy and effectiveness of the immune colloidal gold (ICG) strip developed in this assay, we carried the ICG assay with a panel of control biological samples containing a GPV-positive and a GPV-negative control. The result also can be used as a reference when other samples carried by ICG assay.

### Assay Principle

This assay was based on the immunochromatography and initially developed for determination of pregnancy on spot (Paek et al., 2000). Specific contents are as follows: Two antibodies binding distinct epitopes present on the virus molecule are used. One (coating antibody) labeled with a colloidal gold nanoparticles and the other (capture antibody) fixed onto surfaces of NC membrane. The coating antibody is in a dehydrated state within the conjugate pad. When standard solution or sample were added onto the sample pad of the test strip, the binder can be instantaneously dissolved upon contact with an aqueous medium containing virus. Then the antibody formed a complex with the virus in the liquid phase and moved forward continuously until it was captured by the antibody fixed on the surfaces of NC membrane, which generated a signal in proportion about the virus concentration. Furthermore, an additional antibody specific to the coating antibody can be used to produce a control signal. The absorbent pad is located at the top to induce by capillarity that enables the immune complex to be pulled to the fixed antibody. A visible color appeared in less than 10 min, and the intensity determines the amount of the virus. In other word, the more virus that was present in the sample, the more noticeable the red band appeared.

### Sensitivity, Specificity, and Stability of the Immunochromatographic Strip

The purified GPV suspension which concentrated in the previous work was diluted to different concentrations (1.2 mg/ml, 120 μg/ml, 12 μg/ml, 1.2 μg/ml, 120 ng/ml, 12 ng/ml and 1.2 ng/ml), and PBS (pH 7.4) was used as negative control. ICG strip test, agar diffusion test and PCR test were carried out, respectively, with different concentration solutions, to compare and evaluate the sensitivity of the immunochromatographic strip (Liu et al., 2017).

Common viruses of goose were tested to evaluate the specificity of the immunochromatographic strip, including GPV, avian influenza virus (H5, H7, H9), duck hepatitis virus, tembusu virus, fowl adenovirus, goose reovirus and muscovy duck parvovirus. PBS (pH 7.4) was used as negative control.

All immunochromatographic strips were stored for 8 months at room temperature (25°C) and at 4°C, respectively, to evaluate the stability of the strips during storage (Liu et al., 2017). The sample contaminated by GPV was used as a positive control and PBS (pH 7.4) was used as a negative control.

### Detection of Clinical Samples

A total of 92 suspected samples were used in this study, and clinically determined symptoms including diarrhea and intestinal embolism can be detected. All samples were collected as part of routine veterinary procedures containing goose allantoic fluid and supernatant of tissue homogenate, and they were examined using the developed test strips. The operation was determined according to the method described above. These samples were also examined by the agar diffusion test and PCR test, to compare and analyze the results of these tests with those obtained via ICG strip test.

## Results

### Optimal Concentration of the Coating Antibody and Capture Antibody

**FIGURE 2:**
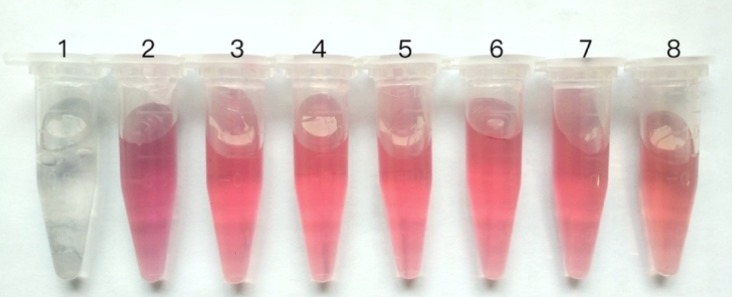
The illustration of immunochromatographic test results.

Concentrated virus suspension was confirmed positive for PCR test. The concentration of GPV suspension was determined to be 1.2 mg/ml and the purity is above 85%, which both verified by SDS-PAGE and analyzed by Quantity One software. Hybridomas were obtained by the fusion of spleen cells from immunized mice with SP2/0 myeloma cells as described above. The results of affinity purification for the selected clones displayed high affinity and no cross-reactivity with related virus molecules. The monoclonal antibody was purified by sequential precipitation and determined to be 5 mg/ml with the DeNovix equipment. For the capture antibody, the minimum Mab concentration labeled with colloidal gold of red invariant is 7.5 μg/ml (**Figure 2**), therefore optimal concentration was determined to be 9 μg/ml according to the methods in Section “Materials and Methods.”

After four immunizations for 5 weeks, the antibody titers in the serum reached 1:256. The polyclonal antibody was purified by sequential precipitation was determined to be 3.2 mg/ml with Bradford method. The purified pAb was diluted into gradient concentration, and the color of the test line deepened gradually as the concentration of pAb increased. When the concentration of pAb reached 1.6 mg/ml, the color of the test line was maintained. Therefore, the optimal concentration of the coating antibody is 1.6 mg/ml.

### Result Verdict and Effectiveness of the Immunochromatographic Strip

**FIGURE 3:**
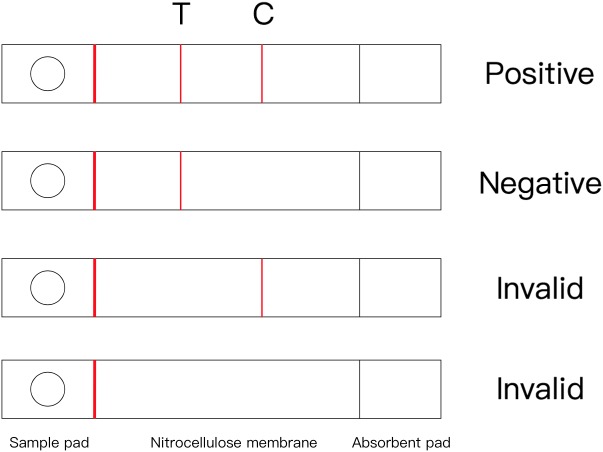
The optimal concentration labeled with colloidal gold of Mab 1: 0 μg/ml, 2: 5 μg/ml, 3: 7.5 μg/ml, 4: 10 μg/ml, 5: 20 μg/ml, 6: 30 μg/ml, 7: 50 μg/ml, 8: negative control.

In this assay, 80–120 μl of samples were added to the sample pad of the immunochromatographic strip, then the strip flat for 5–7 min. As described in **Figure 3**, if the sample contained GPV, the red band appears at the T line and the C line; if the GPV is not present in the sample, the red band appears only at the C line. When there is no red band at the C line, the test results are invalid regardless of whether the red band appears at the T line (Shim et al., 2013).

A panel of control biological samples (positive and negative control for GPV) were examined by ICG strip. The result showed that both two red band appeared when examining the positive control and one red band appeared only at the C line when examining the negative control.

### Sensitivity of the Immunochromatographic Strip

**FIGURE 4:**
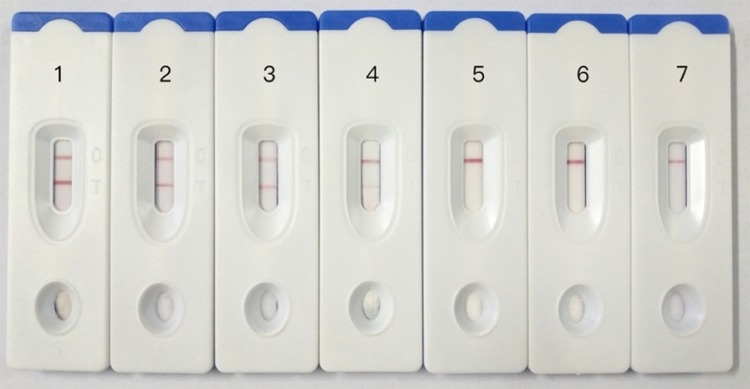
Sensitivity testing of immunochromatographic strip. 1: GPV, 1.2 mg/ml, 2: GPV, 120 μg/ml, 3: GPV, 12 μg/ml, 4: GPV, 1.2 μg/ml, 5: GPV, 120 ng/ml, 6: GPV, 12 ng/ml, 7: GPV, 1.2 ng/ml.

The sensitivity of the ICG assay was evaluated by testing 10-fold serial dilutions of the virus concentration (1.2 mg/ml to 1.2 ng/ml), and the results showed in **Figure 4** that the lowest limit of detection of the assay was 1.2 μg/ml. By comparison, the result of agar diffusion test is 1.2 mg/ml, and PCR test is 1.2 ng/ml.

### Specificity of the Immunochromatographic Strip

**FIGURE 5:**
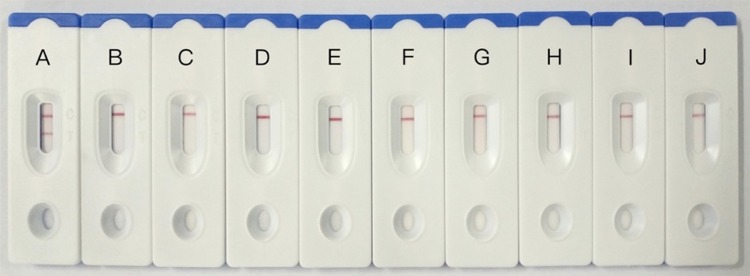
Specificity testing of immunochromatographic strip. **(A)** Goose parvovirus, **(B–D)** avian influenza virus (H5, H7, H9), **(E)** duck hepatitis virus, **(F)** tembusu virus, **(G)** fowl adenovirus, **(H)** goose reovirus, **(I)** muscovy duck parvovirus, **(J)** negative control.

Goose parvovirus and other goose samples including avian influenza virus (H5, H7, H9), duck hepatitis virus, tembusu virus, fowl adenovirus, goose reovirus and muscovy duck parvovirus, were examined by the immunochromatographic strip, and results showed in **Figure 5** that only GPV sample presented two red bands in test and control line and others presented only one red line in control line. These confirmed that the immunochromatographic strip had high specificity for detecting GPV, and no cross-reaction was observed.

### Stability of Immunochromatographic Strip

To determine the stability of the immunochromatographic strip, the ICG assay was carried out with the same batch of strips after stored at 4°C and 25°C for 8 months. The results have been shown in **Table 1**, we can see that the ICG strip could be stored for 4 months at 25°C and for 6 months at 4°C, respectively.

**Table 1:** Stability experiment results.

Storage temperature	Storage time (month)								
	1	2	3	4	5	6	7	8
Room temperature (25°C)	+++	+++	+++	+++	++	+	-	-
Refrigerant temperature (4°C)	+++	+++	+++	+++	+++	+++	+	-

*+++, the valid rate was 100%; ++, the valid rate was 66%; +, the valid rate was 33%; -, the valid rate was 0%.*

### Clinical Sample Application

Immune colloidal gold strips test established in the study were performed on 92 clinical samples of suspected cases of GPV, and comparing the results of these tests with those obtained via agar diffusion test and PCR test. The results are summarized in **Table 2**. The positive rate of goose allantoic fluid was determined to be 88.8% by the ICG strips test and 92.6% by the PCR test, and the coincidence rate of these two methods was 96%. For supernatants of tissue homogenate, it was 84.2% by the ICG strips test and 97.3% by the PCR test, and the coincidence was only 76.4%. For all clinical samples, the coincidence rate was up to 91.9%. Moreover, only few positive samples could be detected by agar diffusion test.

**Table 2:** Ninety-two clinical samples of suspected infected cases were used to detect the repeatability of this method.

Type of samples	Positive sample in the study		
	PCR test	Agar diffusion test	ICG strips test
Goose allantoic fluid	50/54	2/54	48/54
Supernatant of tissue homogenate	37/38	3/38	32/38

## Discussion

Thus far, China is already the largest country of waterfowl population in the world. However, infectious diseases are the biggest obstacle to the expansion and development of this industry, and GPV is one of the most serious viral pathogens. At present stage, prevention and early detection are still effective ways to control disease. Many routine detection methods for GPV had been developed. Specifically, GPV isolations using embryonating goose eggs, or primitive goose embryo fibroblasts are widely used in virology diagnosis. PCR have been effectively applied for the rapid detection since they were developed in the late 1990s. Real-time PCR test was used as a highly sensitive and specific method for quantitatively detecting GPV DNA, and thus can detect this virus. Furthermore, agar diffusion test and Loop-Mediated isothermal amplification were reported for more simple detection of GPV infections. Indirect ELISA based on VP3 protein are commonly used for the detection of GPV antibodies.

The colloidal gold-based immunochromatographic assay was considered to be a more rapid and simple detection method for pathogens in the field. It was widely applied in the specific detection of poultry and other pathogens, such as avian influenza virus, reovirus, mycoplasma suis. However, as we know, no research has yet to use this technique to detect GPV. So, in this study, a colloidal gold-based immunochromatographic assay was developed for the rapid detection of GPV. Compared with previous ICG strip test, this method did not need to block NC membrane and conjugate pad in advance, but there was not a substantial drop-off of specificity and stability. In spite of the sensitivity of the assays developed is lower than that of PCR test, this method is more convenient, requires less equipment and can be completed within 10 min. Furthermore, its sensitivity was significantly higher than that of agar diffusion test.

The results obtained with 92 clinical samples suspected of having GPV infection showed that positive incidence detected by the PCR test was similar with the ICG strip test. Particularly, the PCR test results of goose allantoic fluid were consistent with those of the ICG strip test in all except two cases. The result obtained from samples of supernatants of tissue homogenate was lower, which may be caused by the polyclonal antibody against GPV. In future research, we can develop another monoclonal antibody against GPV to apply as coating antibody.

## Conclusion

It is very suitable for detection of GPV in the field by ICG strips test, and it has an important clinical significance for prevention and detection of pathogens.

## Author Contributions

YoD, YT, and XY conceived and designed the experiments. XY, HC, ZW, and YaD performed the experiments. XY, JY, and XN analyzed the data. XY and YT contributed reagents, materials, and analysis tools. XY and LW wrote the paper. LW promoted the product.

## Conflict of Interest Statement

The authors declare that the research was conducted in the absence of any commercial or financial relationships that could be construed as a potential conflict of interest.
